# Supplementary material for: An open-source framework for large-scale, flexible evaluation of biomedical text mining systems
Source: J Biomed Discov Collab. 2008 Jan 29;3:1. doi: 10.1186/1747-5333-3-1 (PMC2276192; doi:10.1186/1747-5333-3-1)
Supplement: Additional file 1 — Evaluation platform source code. The additional file contains the software evaluation framework discussed in this paper. The most current release can be downloaded from SourceForge.net [1]. See the accompanying README file for instructions on installation and use. Also included in the distribution is a collection of UIMA wrappers for some commonly used BioNLP tools and annotated corpora. [file 1747-5333-3-1-S1.zip › bionlp_uima_repository-1.0.1/docs/api/src-html/edu/uchsc/ccp/uima/util/opendmap/ReferenceProcessor.html]

```
001    /**
002     * The OpenDMAP for Protege Project
003     * May 2006
004     */
005    package edu.uchsc.ccp.uima.util.opendmap;
006    
007    import java.util.ArrayList;
008    import java.util.Collection;
009    
010    import edu.uchsc.ccp.opendmap.Parser;
011    import edu.uchsc.ccp.opendmap.Reference;
012    
013    /**
014     * Get the the desired references that DMAP has recognized.  Order
015     * them according to a simple score that prefers references that
016     * include every word over the span that was used to recognize
017     * them.
018     * 
019     * @author R. James Firby
020     */
021    public class ReferenceProcessor {
022    
023            /**
024             * Get the desired references ordered by score.
025             * 
026             * @param parser The parser that has processed an utterance and is holding the references.
027             * @param interests The references of interest.  A collection of reference types.
028             * @param debug True if detailed debugging information should be printed.
029             * @return The ordered list of references of interest.
030             */
031            public static Collection<Reference> getRecognizedReferences(Parser parser, Collection<String> interests, boolean debug) {
032                    // Grab up any generated output
033                    if (debug) {
034                            // Print out everything we saw
035                            Collection<Reference> all = parser.getReferences();
036                            System.out.println("\nAll references:");
037                            for (Reference r: all) {
038                                    System.out.println(r.getStart() + ".." + r.getEnd() + " " + r.getReferenceString());
039                            }
040                    }
041            
042                    // Find all of the unsubsumed references that were generated
043                    Collection<Reference> references = parser.getUnsubsumedReferences(debug);
044                    if (debug) {
045                            System.out.println("\nUnsubsumed references");
046                            for (Reference r: references) {
047                                    System.out.println(r.getStart() + ".." + r.getEnd() + " " + r.getReferenceString());
048                            }
049                    }
050                    
051                    // Extract and score any interesting references found
052                    ArrayList<Reference> seen = new ArrayList<Reference>();
053                    for (Reference r: references) {
054                            if (isConceptOfInterest(r, interests)) {
055                                    // Sort into list
056                                    boolean done = false;
057                                    for (int i=0; i<seen.size(); i++) {
058                                            Reference ref = seen.get(i);
059                                            if (calculateScore(r) > calculateScore(ref)) {
060                                                    seen.add(i, r);
061                                                    done = true;
062                                                    break;
063                                            }
064                                    }
065                                    if (!done) seen.add(r);
066                            }
067                    }
068                    
069                    // Done
070                    return seen;
071            }
072    
073            /**
074             * Check whether a reference is a concept of interest.
075             * 
076             * @param r The reference to check.
077             * @param interests The collection of concepts of interest
078             * @return True if the reference is a concept of interest
079             */
080            private static boolean isConceptOfInterest(Reference r, Collection<String> interests) {
081                    // If no interests specified, return everything
082                    if ((interests == null) || (interests.size() <= 0)) return true;
083                    // Check whether the reference is an interesting concept
084                    boolean found = false;
085                    for (String interest: interests) {
086                            if (r.isa(interest)) {
087                                    found = true;
088                                    break;
089                            }
090                    }
091                    return found;
092            }
093            
094            /**
095             * Score the reference.
096             * 
097             * @param r The reference
098             * @return The score
099             */
100            public static double calculateScore(Reference r) {
101                    return ((double)(calculateSpan(r) - r.getMissing())) / ((double)(calculateSpan(r)));
102            }
103            
104            /**
105             * Calculate the span of a reference.
106             * 
107             * @param r The reference
108             * @return The span
109             */
110            private static int calculateSpan(Reference r) {
111                    return r.getEnd() - r.getStart() + 1;
112            }
113            
114    }
```
